# Assessment of the Mutagenicity of Sediments from Yangtze River Estuary Using Salmonella Typhimurium/Microsome Assay

**DOI:** 10.1371/journal.pone.0143522

**Published:** 2015-11-25

**Authors:** Li Liu, Ling Chen, Tilman Floehr, Hongxia Xiao, Kerstin Bluhm, Henner Hollert, Lingling Wu

**Affiliations:** 1 Key Laboratory of Yangtze Water environment, Ministry of Education, College of Environmental Science and Engineering, Tongji University, Shanghai, China; 2 Department of Ecosystem Analysis, Institute for Environmental Research (Biology V), ABBt - Aachen Biology and Biotechnology, RWTH Aachen University, Aachen, Germany; 3 College of Resources and Environmental Science, Chongqing University, Chongqing, China; 4 School of Environment, Nanjing University, Nanjing, China; East Carolina University, UNITED STATES

## Abstract

Sediments in estuaries are of important environmental concern because they may act as pollution sinks and sources to the overlying water body. These sediments can be accumulated by benthic organisms. This study assessed the mutagenic potential of sediment extracts from the Yangtze River estuary by using the Ames fluctuation assay with the *Salmonella typhimurium* his (−) strain TA98 (frameshift mutagen indicator) and TA100 (baseshift mutagen indicator). Most of the sediment samples were mutagenic to the strain TA98, regardless of the presence or absence of exogenous metabolic activation (S9 induction by β-naphthoflavone/phenobarbital). However, none of the samples were mutagenic to the strain TA100. Thus, the mutagenicity pattern was mainly frameshift mutation, and the responsible toxicants were both direct (without S9 mix) and indirect (with S9 mix) mutagens. The mutagenicity of the sediment extracts increased when S9 was added. Chemical analysis showed a poor correlation between the content of priority polycyclic aromatic hydrocarbons and the detected mutagenicity in each sample. The concept of effect-directed analysis was used to analyze possible compounds responsible for the detected mutagenic effects. With regard to the mutagenicity of sediment fractions, non-polar compounds as well as weakly and moderately polar compounds played a main role. Further investigations should be conducted to identify the responsible components.

## Introduction

Sediments in estuaries are of important environmental concern because they may act as potential sinks for a multitude of hazardous compounds. These sediments may also act as sources of such pollutants to the overlying water bodies and can be accumulated by benthic organisms. Thus, contaminated sediments in rivers and estuaries may have a potential hazard to the benthic biota and the human health through the food chain.

*In vitro* bioassays are useful in assessing sediment quality. These bioassays can be conducted in a well-controlled and predetermined environment; they can provide rapid results, cost less, and are more reproducible [1]. Mutagenicity has been an increasing concern in recent years because it causes permanent changes in the structure and/or amount of the genetic material of an organism that can lead to heritable changes in its function [2]. *Salmonella*/*microsome* assay is a generally accepted biotest to detect mutagenicity of individual compounds and environmental samples [3]. The assay used the standard tester strains TA98 and TA100. The two different type strains could be applied to detect frame-shift mutations and base-substitution mutations, respectively. In the present study, the Ames fluctuation assay was used to detect the mutagenicity of sediment extracts from the Yangtze River estuary. This assay is an improved version of the conventional Ames test, which used incubated agar plates, whereas the Ames fluctuation assay is performed completely in liquid culture, in which the amount of microorganisms is quantitatively measured using turbidity or pH indicating reagents [4,5,6]. This assay is conducted in accordance with the ISO guideline and has been widely used to assess mutagenic potential of sediments, suspended particulate matter (SPM), and water; it is a qiute useful assay that measures the ability of compounds to cause back mutations in *Salmonella* bacteria [7,8,9,10].

However, biotesting alone does not provide information on the possible related compounds that cause ecotoxicological effects. Therefore, it is not a sufficient basis for risk reduction measures, such as remediation or emission control. Effect-directed analysis (EDA) is a powerful tool that is used to identify toxic substances in complex environmental samples. It can extensively evaluate the toxic potency substances in environmental matrix [11,12]. Several studies have successfully applied EDA to identify toxic chemicals in sediments, such as mutagens and ethoxyresorufin-*O*-deethylase inducers in aquatic sediments from the Neckar catchment area in Germany [13] and endocrine disruptors of sediments from Zierikzee harbor [14]. Lübcke-von Varel et al. [15] applied EDA in the sediment extracts of Bitterfeld. They identified and quantitatively confirmed that dinitropyrenes and 3-nitrobenzanthrone act as major mutagens in that area. Higley et al. [8] used the EDA method to analyze the mutagenic effects of sediment extracts from the upper Danube River in Germany. The EDA of three sediment extracts from the polluted sites of the river Elbe basin suggests that the polar compounds are the dominant substances for the investigated mutagenicity [16].

The Yangtze River estuary at the eastern coast of China is one of the most important industrial and agricultural areas of the country. Given the increasing development of agriculture and industry in this area, lots of organic pollutants enter into the Yangtze River, such as polychlorinated biphenyls (PCBs) [17], polycyclic aromatic hydrocarbons (PAHs) [18], perfluorinated compounds (PFCs) [19], and other emerging contaminants, such as polybrominated diphenyl ethers (PBDEs) [20,21]. Although these organic pollutants have been detected in the Yangtze River estuary for decades, the potential for biological effects in exposed non-target organisms is seldom reported [22,23]. In our previous study in the same sampling area, we applied neutral red retention and 7-ethoxyresorufin-*O*-deethylase assays in the rainbow trout (*Oncorhynchus mykiss*) liver cell line RTL-W1. The results showed that the cytotoxicity and AhR-mediated activity of sediments from the Yangtze River estuary range from low to moderate level compared with the ecotoxicity of sediments from other river systems [24]. The present study constitutes a follow-up approach to investigate the mutagenic potential of sediments in Yangtze River estuary. The aims are to (1) assess the mutagenicity of the surface sediment samples from the Yangtze River estuary using the Ames fluctuation assay with bacteria strains TA98 and TA100 and (2) apply the concept of EDA to identify the potential mutagenic components through fractionation of sediment extracts.

## Materials and Methods

### Sample collection

**Fig 1:**
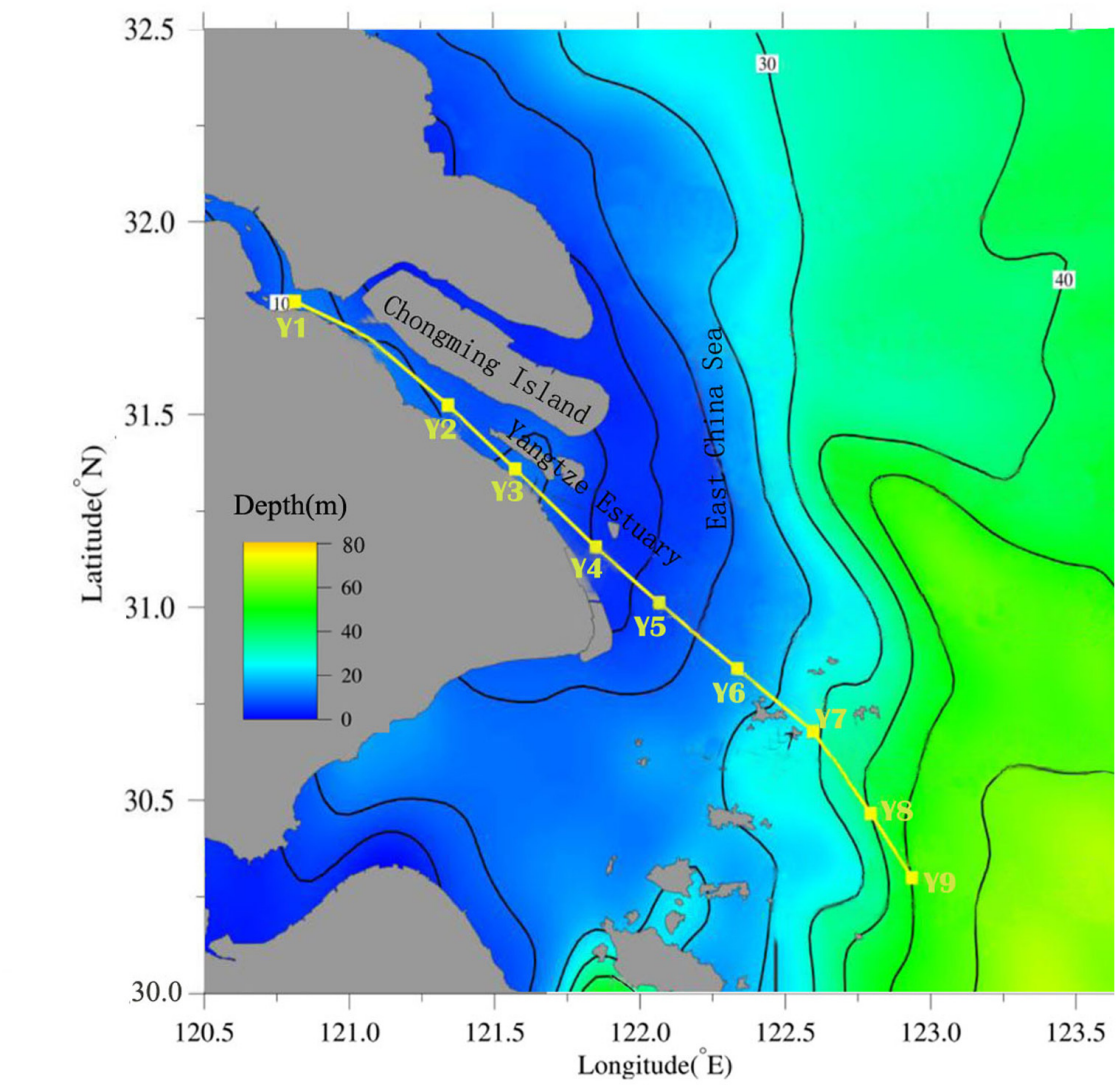
Map of the sampling locations in the Yangtze River estuary.

Surface layers of sediment samples (0–5 cm) were collected from nine locations of the Yangtze River estuary in March 2012 using a stainless steel grab sampler. Locations of the sample sites are shown in Fig 1. The details of the sampling information are available elsewhere [24]. The sampling locations were collected along the salinity gradient of the estuary. Samples Y1 to Y3 were fresh water dominated sediments. Sites Y4 and Y5 were located in the turbidity maximum zone and the samples were brackish water dominated sediments. Sites Y6 to Y9 were located in the river plume zone and the samples were marine sediments. All samples were transported to the laboratory and stored at −20°C until further analyses. Samples were freeze-dried at −50°C, passed through a 100-mesh screen (150 μm), and stored in combusted glass with Teflon-lined lids at −20°C in the dark until further analysis [25].

### Sediment crude extract procedure

Detailed descriptions of sample preparation have been published previously [24,25]. In brief, 20 g of the dried sediment samples was separately extracted with acetone (Merck, HPLC) for 48 h using standard reflux (Soxhlet) extractors at approximately six cycles per hour. The extracts were reduced to approximately 2 mL by using a rotary evaporator. One half of the sample was re-dissolved in 1 mL of dimethylsulfoxide (DMSO) (Sigma, Deisenhofen, Germany) for *in vitro* biotests, resulting in final concentrations of 10 g sediment dry weight per mL DMSO (10 g/mL). The other subsample was re-dissolved in 1 mL of n-Hexane (Merck, HPLC) for multilayer fractionation, which was used for the EDA analysis to identify unknown mutagens in the sediments [26], resulting in final concentrations of 10 g sediment dry weight per mL n-Hexane (10 g/mL). Extracts were stored at −20°C until further analysis.

### Multilayer fractionation procedure

Sediments used for fractionation were selected on the basis of the maximum mutagenic potential detected in the crude extracts. Multilayer fractionation was performed according to previously described methods [24]. Sediment extracts were eluted to non-polar paraffinic components (F1), weakly and moderately polar components (F2) and more polar components (F3) with *n*-hexane (Merck, HPLC), n-hexane/dichloromethane (Merck, HPLC; 7/3, v/v) and acetone/methanol (Merck, HPLC; 1:1, v/v), respectively. The eluates were initially reduced to 2 mL using a rotary evaporator and further evaporated to near dryness under a gentle nitrogen stream. The final concentration was set to 10 g/mL DMSO. Fractions were stored at −20°C in darkness until analysis.

### Ames fluctuation assay

All crude sediment extracts and fractions were analyzed in the Ames fluctuation assay according to the protocol given by Reifferscheid et al. [5]. The assay used *S*. *typhimurium* strains TA98 and TA100 with or without exogenous metabolic activation (S9 induction by β-naphthoflavone/phenobarbital) to measure frameshift mutations and base pair substitutions, respectively. The extracts were serially diluted with DMSO on each plate to yield a concentration range from 200 mg to 6.3 mg sediment equivalents per mL test medium. Each extract was tested in triplicate in each concentration. Details about the assay procedure were described previously [8]. Prior to use in the assay, the bacterial strains were grown overnight with shaking at 37°C. The bacteria were diluted in exposure medium and exposed to sediment extracts, 2% DMSO solvent control, and positive control. Subsequently, the plates were incubated at 37°C with shaking for 100 min. A bromocresol purple indicator medium (2.5 mL) was then added to each well. Transfer 50 μL from the 24-well plates (TPP, Trasadingen, CH) into the 384-well plates (Greiner Bio-one) for controls and samples. The volume of one 24-well plate is sufficient for 3 replicates in one 384-well plate. The plates were incubated at 37°C for 48 h. The number of yellow wells per replicate group was counted and compared with the solvent control. The maximum induction factors (IF_max_) were computed, which give the induction of the highest inducing sample concentration referred to the negative control induction.

### Chemical analysis of total organic carbon (TOC)

The TOC was determined with the Shimadzu TOC-VCPN with solid sample module (SSM-5000A; Shimadzu, Japan). The overall standard deviation of measurements was less than 3% (n = 3).

### Statistical analysis

Data were expressed as mean ± SD. All statistical analyses were performed using SPSS 17.0 (SPSS Inc., Chicago, IL, USA). Homogeneity of variance was assessed with the Levene’s test. Normality of data distributions was assessed by Shapiro–Wilk’s test. ANOVA was used to compare the results of whole extracts of sediment in the Ames fluctuation assay to the controls. The Williams multiple sequential t-test was performed to analyze the differences of all samples from controls. *p* < 0.05 was considered statistically significant.

## Results and Discussion

### Mutagenicity of crude sediment extracts

**Fig 2:**
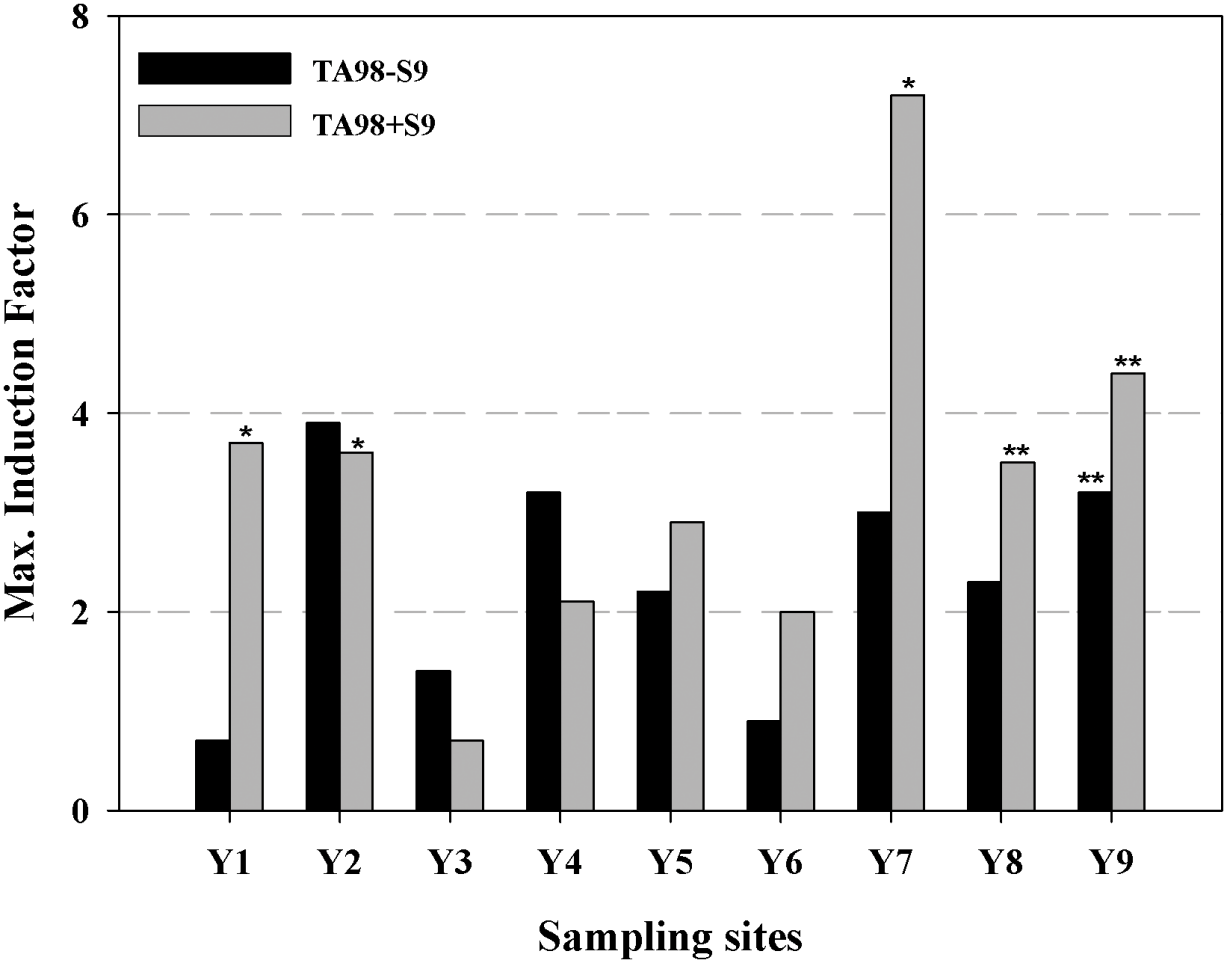
Mutagenic activity of nine sediment extracts from Yangtze River estuary. Mutagenic measured by the Ames fluctuation assay using both TA98 and TA100 bacteria strain with and without bioactivation enzymes (S9). TA100 with or without S9 is not shown because no mutagenic effects were observed in any of the samples. Data are shown as maximum induction factor (IF_max_) as the highest IF score of a particular sample within the dose-response curve. Multiple symbols indicate different significant levels relative to the negative control (NC): *p<0.05, **p<0.01.

The mutagenicity of sediment extracts was investigated with the Ames fluctuation assay using the tester strains TA98 and TA100 with and without metabolic activation S9. Fig 2 and S1 Table showed the results of the tester strain TA98 expressed as IF_max_ values. The mutagenic effects in the bacteria strain TA98 were observed in most of the sediment extracts, regardless of the presence of S9. The results indicate the presence of potential mutagenic compounds, which did not need metabolic activation. As shown in Fig 2, the sediment extracts from site Y7 exhibited the highest mutagenic potential among the samples in the bacteria strain TA98 with S9 (IF_max_ value 7.2). In addition, significant mutagenic effects were observed in TA98 with S9 when exposed to sample extracts of Y1, Y2, and Y9, with IF_max_ values of 3.7, 3.6, and 4.4, respectively. Sample site Y2 caused the most mutagenic potential in the bacteria strain TA98 without S9 (IF_max_ value 3.9), followed by sample sites Y4, Y9, and Y7, with IF_max_ values of 3.3, 3.2, and 3.0, respectively. Overall, most of the samples elicited stronger mutagenic effects in the TA98 strain if S9 was added (except Y2 and Y4). Sample site Y3 showed very low mutagenic potency regardless of S9 addition.

In contrast to the bacteria strain TA98, the sediment extracts did not cause a significant increase in revertants in the bacteria strain TA100, regardless of the presence of bioactivation enzyme S9 (data not shown). Although both the *Salmonella* strains TA98 and TA100 were suitable for the detection of mutagenic potential in the Yangtze River estuary sediment extracts, they showed considerable variation in sensitivity. The different levels of mutagenicity displayed by the two strains may be due to the differences in the type of induced genotoxicity. The strain TA98 usually detects frame shift mutations, whereas the strain TA100 detects base substitution mutations [3]. The current study indicated that the mutagenic potential of the estuarine sediment samples was manifested in the bacteria’s genome by frameshift mechanism (strain TA98). Wu et al. [22] found that the water samples from the Yangtze River estuary exhibit mutagenicity when tested with the strain TA98. However, they detected no mutagenic activity in all samples with the strain TA100. Other research also reported that frameshift mutagens are particularly responsible inducers in the Ames test, when testing water samples of other rivers in China [27,28,29]. The same observation was found with sediment extracts from the German Rhine River [30] and the upper Danube River in Germany [8]. These studies showed that significant mutagenic effects was only observed with the strain TA98 and after metabolic activation, which are consistent with the results of this study. The present study showed that the frameshift mutation was the main mutagenicity pattern for the investigated environmental samples.

Table 1 shows the results of the chemical analysis. In this study, the TOC content of the surface sediment samples ranged from 0.36% to 1.5%. The content of priority pollutant PAHs in the sediment samples has been previously determined, and the concentrations of total PAHs range from 21.5 to 190.5 ng/g dw sediment [24]. Marvin et al. [31] applied a bioassay-directed analysis method to identify the compounds which responsible for mutagenicity in the Randle Reef sediment extract, and found that Benzo[a]pyrene, benzo-fluoranthenes, indeno[1,2,3-cd]pyrene, benzo[g,h,i]perylene, and dibenz[a,h]anthracene were the major mutagens. The research of Marvin et al. [32] also showed that polar polycyclic aromatic compounds are potent mutagens that require reductive metabolic activation. Other investigations demonstrated that PAHs can account for more than 10%–20% of the overall mutagenic activity and suggested that various organic compounds may be responsible for the genotoxic effects [33,34]. However, the study of Rhine River sediments found no correlation between the analyzed priority PAHs and the corresponding biotests [30]. In the present study, a weak correlation was observed between the PAH contents and the mutagenicity of the strain TA98 with S9 (r = 0.12, *p* > 0.05). This result indicates that other pollutants in the extracts may cause the mutagenicity of the Yangtze River estuary sediment.

**Table 1:** Content of total organic carbon (TOC) (%) and concentrations of the 16 US EPA-polycyclic aromatic hydrocarbons (PAHs) (ng/g dw) in sediment samples from the Yangtze River estuary.

Sampling site	Y1	Y2	Y3	Y4	Y5	Y6	Y7	Y8	Y9
TOC (%)	0.7	0.4	1.5	1.3	1.3	1.5	1.5	1.4	0.8
Naphthalene	6.0	47.0	11.0	22.0	7.0	4.0	26.0	7.0	8.0
Acenaphthylene	n.d.	4.0	n.d.	1.0	2.0	1.0	2.0	2.0	2.0
Acenaphthene	n.d.	2.0	n.d.	2.0	2.0	n.d.	n.d.	1.0	2.0
Fluorene	1.0	5.0	1.0	3.0	6.0	2.0	3.0	4.0	4.0
Phenanthrene	2.0	15.0	1.0	14.0	12.0	5.0	6.0	11.0	9.0
Anthracene	2.0	4.0	1.0	3.0	4.0	1.0	2.0	3.0	2.0
Fluoranthene	2.0	17.0	1.0	20.0	13.0	4.0	6.0	10.0	7.0
Pyrene	1.0	8.5	0.5	10.0	7.0	2.5	3.0	5.5	4.5
Benzo[a]anthracene	1.0	13.0	1.0	14.0	10.0	3.0	5.0	8.0	6.0
Chrysene	2.0	14.0	1.0	13.0	11.0	3.0	5.0	9.0	6.0
Benzo[b]fluoranthene	2.0	19.0	1.0	16.0	18.0	6.0	9.0	14.0	12.0
Benzo[k]fluoranthene	1.0	5.0	n.d.	6.0	5.0	1.0	3.0	4.0	3.0
Benzo[a]pyrene	n.d.	14.0	1.0	13.0	10.0	n.d.	4.0	9.0	6.0
Indeno[1,2,3-cd]pyrene	1.0	10.0	1.0	9.0	8.0	2.0	4.0	7.0	5.0
Dibenz[a,h]anthracene	n.d.	3.0	n.d.	2.0	2.0	n.d.	1.0	2.0	1.0
Benzo[g,h,i]perylene	1.0	10.0	1.0	8.0	8.0	2.0	4.0	7.0	5.0
**Sum of EPA-PAHs**	**22.0**	**190.5**	**21.5**	**156.0**	**125.0**	**36.5**	**83.0**	**103.5**	**82.5**

Note: The data of PAHs were obtained from Liu et al. (2014). n.d. = not detectable or below the detection limit.

The Yangtze River estuary is one of the largest worldwide with rapid economic development, a number of petroleum and chemical plants, and harbor. Numerous industrial wastewater and domestic sewage are discharged in this region and may act as potential mutagenic pollution sources. This area was a mixed pollution zone and the concentration of the pollutants may distribute uneven in different area. The runoff and the particle size of the sediment samples may also influence the distribution of the pollutants. Thus, the combination of these factors may cause to the mutagenicity varied among different sites. In this study region, several organic pollutants, such as PCBs and PFCs, which have been confirmed to possess mutagenic or genotoxic properties and can cause severe effects on health (e.g., cancer formation), were detected in the study region [35,36]. The concentration of PCBs in SPM of the Yangtze River estuary ranged up to 51 ng/g [37]. The concentrations of PFOS ranged from 73 ng/g to 537 ng/g in the South Branch of the Yangtze River estuary [38,39].

Associating the concentration of mutagenic/genotoxic chemicals analyzed in this study to the measured mutagenicity is difficult. Numerous studies that combine chemical and biological approaches for hazard assessment of complex environmental mixtures indicate that the priority pollutants often play a poor role in toxicity [13,40]. Several EDA studies showed that a major portion of the mutagenic and genotoxic activities of sediment extracts is caused by non-priority pollutants, such as methylbenzo[e]pyrene and methylperylene [13,34]. In our previous report, we applied the concept of EDA and found that the priority PAHs seem only responsible for a minor portion of the total AhR-mediated activities [24]. The results of these studies suggest that hazard assessment of environmental matrices should not only focus on priority pollutants but also need to consider the key toxic pollutants.

### Mutagenicity in multilayer fractions of crude sediment extracts

**Fig 3:**
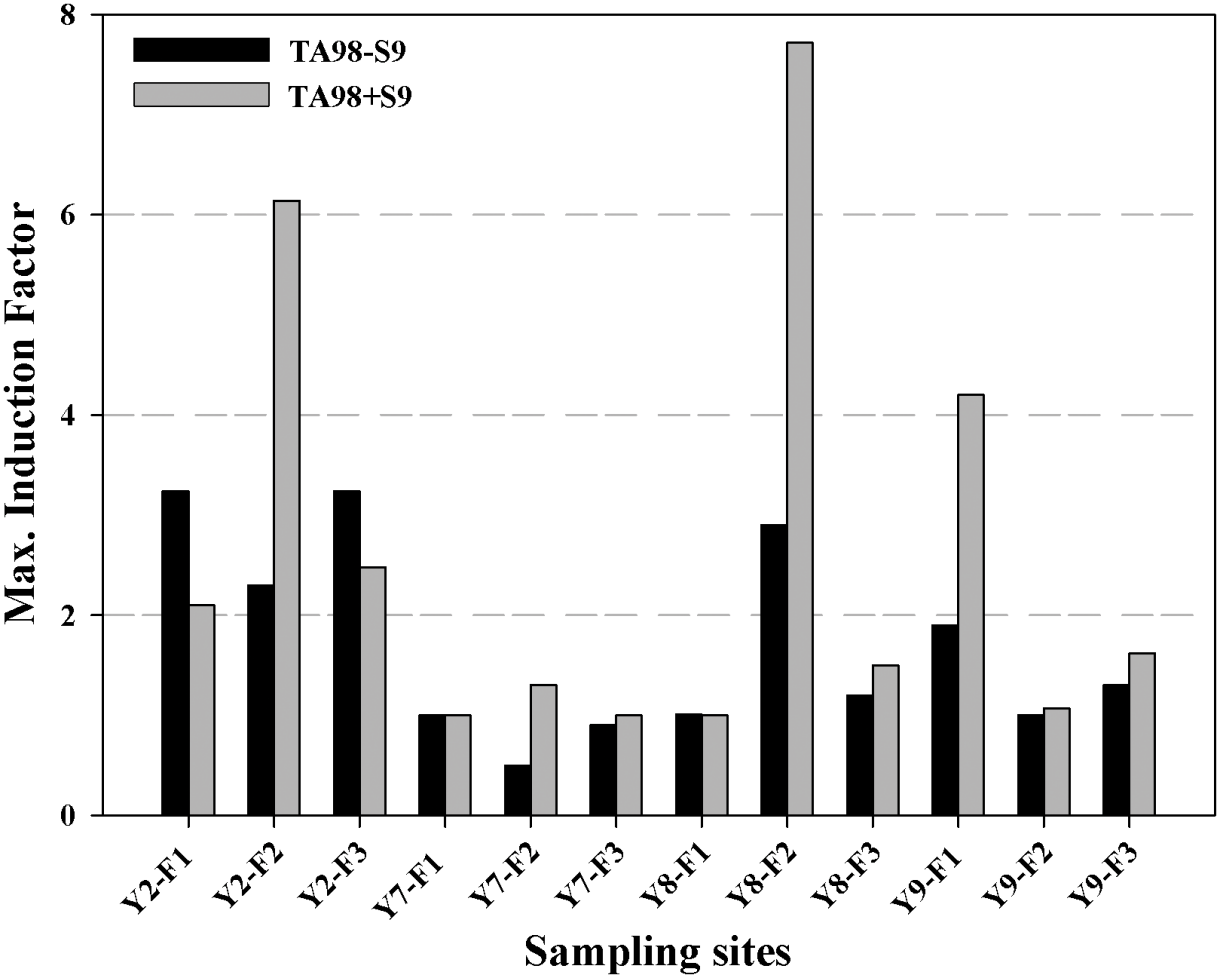
Mutagenic activity of three fractions of samples Y2, Y7, Y8 and Y9. Mutagenicity measured by the Ames fluctuation assay using TA98 bacteria with and without bioactivation enzymes (S9). Mutagenic activity is expressed as maximum induction factor within the dose-response curve.

According to the results of the Ames fluctuation assay with crude extracts (Fig 2), the sediment samples from sites Y2, Y7, Y8, and Y9, which possessed the highest induction factors, were selected for fractionation into three components according to polarity. Subsequently, the mutagenicity of the multilayer fractions was detected. All fractions were only tested with strain TA98. Fig 3 and S2 Table showed the mutagenic activity caused by the multilayer fractions (F1 to F3) of each sample. The results were given as IF_max_. As shown in Fig 3, the mutagenic potential of the different fractions evidently varied. In general, all of the three fractions showed mutagenicity in the strain TA98 with and without S9 activation for site Y2. Weakly and moderately polar components (F2) showed the strongest mutagenicity with the tester strain TA98 with S9, and the IF_max_ value was 6.1. This value was much higher than that of the crude extracts of site Y2. For site Y8, only fraction F2 showed strong mutagenicity with and without S9. The value was also much higher than that of the corresponding crude extracts. However, for site Y7, the crude extract indicated strong mutagenic potential, but very low potency occurred in all fractions in the strain TA98 with or without S9. For site Y9, only fraction F1 showed relative high mutagenicity with S9.

In general, the study found that non-polar components as well as weakly and moderately polar components yielded stronger mutagenicity than the more polar ones. The research by Vahl et al. [41], who reported that less polar extracts displayed higher mutagenic activity values than the methanol extract values of sediments from the River Elbe (Germany) by using the Ames test. Similar results of Brack et al. [13] indicated that the nonpolar aromatic fraction and the moderately polar fraction were significantly mutagenic with and without S9 activation in the Neckar river basin (Germany), whereas the aliphatic fraction and the very polar fraction did not exhibit mutagenicity. The study of Lübcke-von Varel et al. [42] found that the moderately polar fractions present stronger mutagenicity than the polar fractions of sediments from the Elbe river basin. In contrast to the results of this study, Picer et al. [43] found that coastal Adriatic sediments (Croatia) contaminated with industrial and municipal waste water have detectable mutagenic activity (TA98 with S9), which was primarily attributed to polar compounds. Wölz et al. [10] showed that the SPM at River Rhine indicates more polar fractions as mutagenic active with SPM sampled after the discharge peak (IF_max_ = 14.7). The study of Higley et al. [8] showed that sediment fractions which contain more polar compounds induce significant mutagenic effects at all sites from the upper Danube River in the bacteria strain TA98 without S9. The difference among the results of these studies may be attributed to the varied genotoxic pollutants in these sample extracts [44]. Consequently, further studies should be focused on identifying possibly mutagens.

In addition, the level of mutagenicity in the present study is different among the crude extracts and fractions. This result may be due to the interactions of the different substances changed the toxic levels of the matrices, such as synergistic or antagonistic effects [45]. These interactions of different substances contained in the sediments could result in mutagenic activity in a single fraction exceeding that of the crude extract, such as fraction F2 from sites Y2 and Y8, as well as fraction F1 from site Y9. This finding indicates the presence of compounds that inhibit mutagenicity or the enzymatic activation of the mutagens in the crude sediment extracts. These antagonistically acting substances may have been separated in the elution process during fractionation, and then mutagenic compounds could exhibit their activity. The multilayer fractionation in the present study was performed with the silica gel/aluminum oxide column. Alumina adsorption appears to be an effective cleanup for subsequent mutagenicity testing [16]. The phenomenon of suppressive effects of chemicals in mixture on the *Salmonella* plate test response in the absence of apparent toxicity have already been determined [46]. In the earlier study by Liu et al. [24], cytotoxicity of Y8 and Y9 was observed. It seems that the crude extracts contained cytotoxic components were cleaned off during the multilayer fractionation and the mutagenic ones elicited their full activity.

In this study, several fractions displayed lower or no mutagenicity compared with the crude extracts, especially in the fractions of site Y7. Clear dose dependency was not observed for mutagenicity with and without S9 activation in all the three fractions. This result indicates that the combined activity among the substances may add up to the detected mutagenic responses in the crude extracts. The concept of effect additively could be applied in this occasion. The synergistic or additive mechanisms, which may occur in such complex mixtures, could influence the genotoxic responses [34]. The present study showed that the decreasing component complexity caused the reduced mutagenicity of the mixture, since the mutagenicity was not observed in several fractions.

## Conclusions

The present study showed that the sediment from the Yangtze River estuary exhibited mutagenicity in the tester strain TA98 with and without metabolic activation. Mutagenicity was not found in the strain TA100. A weak correlation was observed between the detected PAHs and the mutagenicity of the sediments. Several non-detected pollutants may contribute to the mutagenicity. Further investigations into the quality of the sediments should include the determination of concentrations of priority and non-priority pollutants. Results of the fractionation showed that non-polar components as well as weakly and moderately polar components play a main role in mutagenicity. Responsible pollutants should be identified in the future.

## Supporting Information

S1 Table Mutagenic activity of nine sediment extracts from Yangtze River estuary. Mutagenic measured by the Ames fluctuation assay using TA98 bacteria strain with and without bioactivation enzymes (S9). Data are shown as maximum induction factor (IF_max_) as the highest IF score of a particular sample within the dose-response curve.
(DOCX)

S2 Table Mutagenic activity of three fractions of samples Y2, Y7, Y8 and Y9. Mutagenicity measured by the Ames fluctuation assay using TA98 bacteria with and without bioactivation enzymes (S9). Mutagenic activity is expressed as maximum induction factor within the dose-response curve.
(DOCX)
