# Supplementary material for: Assessment of the Mutagenicity of Sediments from Yangtze River Estuary Using Salmonella Typhimurium/Microsome Assay
Source: PLoS One. 2015 Nov 25;10(11):e0143522. doi: 10.1371/journal.pone.0143522 (PMC4659643; doi:10.1371/journal.pone.0143522)
Supplement: S1 Table — Mutagenic measured by the Ames fluctuation assay using TA98 bacteria strain with and without bioactivation enzymes (S9). Data are shown as maximum induction factor (IFmax) as the highest IF score of a particular sample within the dose-response curve. (DOCX) [file pone.0143522.s001.docx]

**S1 Table. Mutagenic activity of nine sediment extracts from Yangtze River estuary.** Mutagenic measured by the Ames fluctuation assay using TA98 bacteria strain with and without bioactivation enzymes (S9). Data are shown as maximum induction factor (IFmax) as the highest IF score of a particular sample within the dose-response curve.

| Sampling sites | TA98-S9 | TA98+S9 |
| --- | --- | --- |
| Y1 | 0.7 | 3.7  3.6  0.7  2.1  2.9  2.0  7.2  3.5  4.4 |
| Y2 | 3.9 |  |
| Y3 | 1.4 |  |
| Y4 | 3.2 |  |
| Y5 | 2.2 |  |
| Y6 | 0.9 |  |
| Y7 | 3.0 |  |
| Y8 | 2.3 |  |
| Y9 | 3.2 |  |
