# Supplementary material for: Assessment of the Mutagenicity of Sediments from Yangtze River Estuary Using Salmonella Typhimurium/Microsome Assay
Source: PLoS One. 2015 Nov 25;10(11):e0143522. doi: 10.1371/journal.pone.0143522 (PMC4659643; doi:10.1371/journal.pone.0143522)
Supplement: S2 Table — Mutagenicity measured by the Ames fluctuation assay using TA98 bacteria with and without bioactivation enzymes (S9). Mutagenic activity is expressed as maximum induction factor within the dose-response curve. (DOCX) [file pone.0143522.s002.docx]

**S2 Table. Mutagenic activity of three fractions of samples Y2, Y7, Y8 and Y9.** Mutagenicity measured by the Ames fluctuation assay using TA98 bacteria with and without bioactivation enzymes (S9). Mutagenic activity is expressed as maximum induction factor within the dose-response curve.

| Sampling sites | TA98-S9 | TA98+S9 |
| --- | --- | --- |
| Y2-F1 | 3.2 | 2.1  6.1  2.5  1.0  1.3  1.0  1.0  7.7  1.5 |
| Y2-F2 | 2.3 |  |
| Y2-F3 | 3.2 |  |
| Y7-F1 | 1.0 |  |
| Y7-F2 | 0.5 |  |
| Y7-F3 | 0.9 |  |
| Y8-F1 | 1.0 |  |
| Y8-F2 | 2.9 |  |
| Y8-F3 | 1.2 |  |
| Y9-F1 | 1.9 | 4.2 |
| Y9-F2 | 1.0 | 1.1 |
| Y9-F3 | 1.3 | 1.6 |
